# Determination of a cooling-rate frame for antibiotic-free preservation of boar semen at 5°C

**DOI:** 10.1371/journal.pone.0234339

**Published:** 2020-06-09

**Authors:** Aline F. L. Paschoal, Anne-Marie Luther, Helen Jäkel, Kathi Scheinpflug, Kristin Mühldorfer, Fernando P. Bortolozzo, Dagmar Waberski

**Affiliations:** 1 Unit of Reproductive Medicine of the Clinics, University of Veterinary Medicine, Hannover, Germany; 2 Animal Science Department, Swine Sector, Federal University of Rio Grande do Sul, Porto Alegre, Brazil; 3 Department of Wildlife Diseases, Leibniz Institute for Zoo and Wildlife Research, Berlin, Germany; University of Florida, UNITED STATES

## Abstract

Hypothermic storage of boar semen provides the possibility to omit antibiotics from semen extenders so long as sperm quality is maintained and bacterial growth prevented. The objective of this study was to determine an optimal cooling-rate frame for boar semen preserved at 5°C in an antibiotic-free extender. Semen from eight boars extended in AndroStar^®^ Premium was cooled from 30°C to 5°C using seven different cooling rates, ranging initially from 0.01 to 0.36°C min^–1^ and reaching 5°C between 2 h and 24 h after dilution. Sperm motility, membrane integrity, membrane fluidity, mitochondrial membrane potential and the response to the capacitation stimulus bicarbonate remained at a high level for 144 h at 5°C when the semen was initially cooled in a cooling-rate frame ranging from 0.01 to 0.09°C min^‑1^ in the temperature zone from 30 to 25°C, followed by 0.02 to 0.06°C min^–1^ to 10°C and 0.01 to 0.02°C min^‑1^ to the final storage temperature. A cooling rate of 0.07°C min^–1^ in the temperature zone from 30 to 10°C led to a reduced response to bicarbonate (P < 0.01) and fast cooling to 5°C within 1 h with a cooling rate of 0.31°C min^–1^ resulted in lower values (P > 0.05) of all sperm parameters. In a further experiment, slow cooling with a holding time of 6 h at 22°C induced after 6 h storage a temporary increase in *Escherichia coli* of 0.5 × 10^3^ to 2.4 × 10^3^ CFU mL^–1^ in the sperm-free inoculated extender. Overall, the load of mesophilic bacteria in the stored semen was below 6 × 10^3^ CFU mL^–1^, a level that is not regarded as critical for sperm quality. In conclusion, appropriate cooling protocols were established for the antibiotic-free storage of boar semen at 5°C, allowing the application of hypothermic preservation in research and in artificial insemination.

## Introduction

Artificial insemination (AI) in pigs is a highly efficient breeding technology used worldwide and currently applied in more than 90% of the sows in the main pork-producing countries. Despite the merits of AI in promoting herd health and genetic progress, there is an increasing concern about the role of antibiotics in boar semen extenders in the development of antimicrobial-resistant bacteria and the global spread of resistance genes [1]. Currently, the addition of antibiotics is mandatory in many countries, but antimicrobial alternatives are under research [2,3]. To counteract increasing antimicrobial resistance, efforts have been developed to reduce bacterial load during semen collection, processing and storage [4,5]. The reduction of the conventional storage temperature of boar spermatozoa from 17°C to 5°C was recently proposed as a novel concept to abolish the use of antibiotics in semen extenders [6]. Beside the bacteriostatic effect of low-temperature storage, there is an increasing interest in the preservation of boar semen at 5°C to facilitate temperature stabilization during the transport of semen from the AI center to the sow farm, especially when environmental temperatures are unstable or extreme. However, cold damage to boar spermatozoa is an important concern during storage at such low temperatures [7,8], and it also precludes the large-scale use of cryopreserved boar semen. Compared to other species, boar sperm’s sensibility to chilling injury is associated with a low sterol/phospholipid ratio and a thermotropic phase transition in membrane lipids between 30°C and 10°C [9, 10].

It is well established that delayed cooling increases the resistance of sperm to cooling stress [11], although the underlying mechanisms are only partially known. Holding the diluted semen for up to 24 h at a temperature above 15°C stabilizes the lipid architecture of the plasma membrane [12] and increases the phosphorylation levels of serine residues of several sperm proteins, including heat-shock protein 70 [13]. Holding times have therefore been implemented in boar sperm cryopreservation protocols and have also been recommended for sperm protection in 5°C storage [12]. Extended holding times above 15°C, however, may favor bacterial growth, and thus frustrate antibiotic-free storage strategies at supra-zero temperatures. Hence, ideally, cooling rates would balance the risks of chilling injury and bacterial growth. From this perspective, the optimal cooling rates for boar semen to be stored at 5°C have not yet been reported. For the further development and incorporation of the hypothermic storage concept into AI practice, determining a cooling-rate frame that concurrently is tolerated by spermatozoa and inhibits bacterial growth is required. Therefore, the objective of this study was to determine upper and lower cooling-rate limits for chilling boar spermatozoa in a protective antibiotic-free extender to the desired storage temperature of 5°C. Sperm functionality was assessed with sensitive flow cytometric assays and bacterial growth during storage up to 144 h was considered.

## Materials and methods

### Reagents and media

Chemicals were of analytical grade and, unless otherwise stated, purchased from Sigma-Aldrich (Steinheim, Germany). Fluorochromes were obtained from Life Technologies (Darmstadt, Germany), and semen extender media were obtained from Minitüb (Tiefenbach, Germany).

### Animals and semen processing

A total of eight mature, fertile boars of different breeds (Landrace and Pietrain), under routine semen collection for the production of semen doses, were used. The boars were housed in individual pens with free access to water. All procedures involving animals were performed according to the European Commission Directive for Pig Welfare and were approved by the Institutional Animal Welfare Committee of the University of Veterinary Medicine, Hannover. Semen was collected by the gloved-hand method by trained personnel from the University service dedicated to animal care. At collection, the pre-spermatic phase was discarded and the gel fraction removed by gauze filtration, so that the used ejaculates consisted of the sperm rich and the sperm poor fractions. Normospermic ejaculates only (a minimum of 75% morphologically normal spermatozoa, at least 70% total sperm motility, and total number ≥30 × 10^9^ spermatozoa per ejaculate) were isothermally (32°C) diluted in one step with antibiotic-free AndroStar^®^ Premium extender (Minitüb, Germany) to 2.0 × 10^6^ spermatozoa mL^–1^ in a final volume of 90 mL. Semen doses were then cooled to 5°C as described below and stored at 5 ± 1°C in the dark in a temperature-controlled refrigerator (14160/0352, Minitüb, Germany).

### Semen packaging and cooling

**Fig 1:**
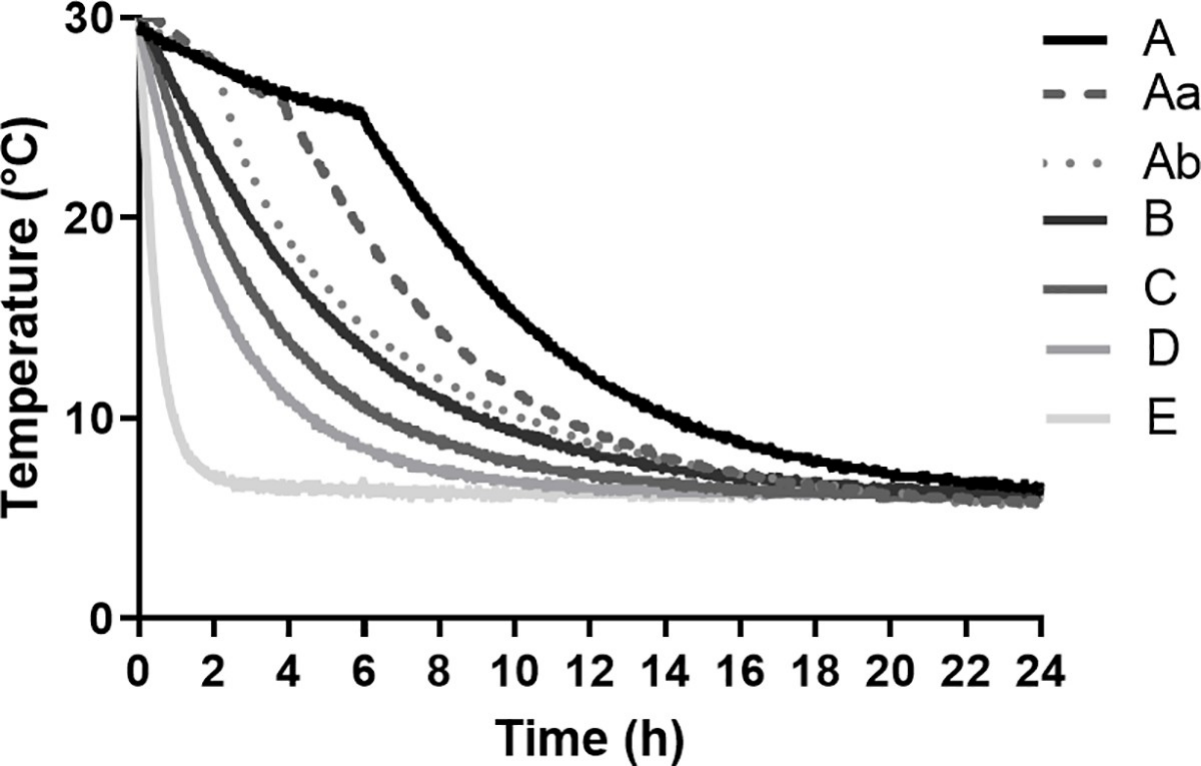
Graphic presentation of the temperature curves used in Experiment 1 (A, B, C, D and E) and Experiment 2 (A, Aa, Ab, B) for cooling semen doses from 30 ± 1°C to 5 ± 1°C). Samples cooled by Curve A, B, C, D and E had an initial cooling rate of 0.01, 0.06, 0.09, 0.14 and 0.65°C min^–1^, respectively, until reaching 25°C. Then, samples were cooled to 10°C at the rates of 0.03, 0.03, 0.05, 0.06 and 0.31°C min^–1^, respectively. Finally, samples were cooled to 5°C at a rate of 0.01°C min^–1^ (Curves A, B, C and D) and at 0.02°C min^–1^ (Curve E). Samples cooled by Curves Aa and Ab had an initial cooling rate of 0.04 and 0.02°C min^–1^, respectively, until reaching 25°C. Samples were then cooled to 10°C at a rate of 0.04°C min^–1^. The final cooling from 10°C to 5°C was performed with cooling rates of 0.01°C min^–1^. The corresponding exponential regressions are described in Table 1.

Cooling curves as shown in Fig 1 were obtained by variations of semen packaging and holding times. Freshly extended semen was added to tubes (Quick Tip Flexitubes^^®^^, Mintüb) to a volume of 90 mL and placed in the center of cardboard boxes surrounded by water-filled tubes. The temperature of all filled tubes was 30°C, corresponding to the temperature of the freshly extended semen. Cooling Curves A, Aa, Ab and B were obtained with 35 tubes in a cardboard box (31.5 × 21.5 × 10.0 cm) and the use of different holding times at 22°C before storage at 5°C. Holding times: Curve A, 6 h; Curve Aa, 4 h; Curve Ab, 2 h; and Curve B, 0 h. Cooling Curve C was obtained with 13 filled tubes in a cardboard box (21.5 × 15.0 × 10 cm), and cooling Curve D was realized with six filled tubes. To obtain cooling Curve E, a single semen tube without packaging was placed into the 5°C storage cabinet directly after semen dilution. For cooling Curves B, C, D and E, no holding times were applied.

Temperature recording during cooling was performed with a multiple-channel datalogger (Mikromec^®^ Multisens MLm 424, Technetics GmbH, Freiburg, Germany) equipped with a flexible sensor positioned in the center of a tube filled with water. This tube was placed inside a box in the central position. The temperature was recorded every 1 min for 24 h. An extra-flexible sensor was placed outside the box to record the temperature of the refrigerator. The respective cooling curves are presented in Fig 1, and the exponential regressions are described in Table 1.

**Table 1:** Exponential regressions describing cooling curves used in Experiment 1 (A, B, C and D, and E) and Experiment 2 (A, Aa, Ab and B) for freshly extended semen in 90 mL tubes. Samples were cooled from 30 ± 1°C to the desired storage temperature of 5 ± 1°C. The corresponding curves are presented in Fig 1.

Cooling	Temperature zone used to calculate each exponential regression		
Rate	30°C to 25°C	25°C to 10°C	10°C to 5°C
A*	y = 29.2 e^-4E-04x^	y = 24.5e^-0,002x^	y = 9.6e^-7E-04x^
Aa*	y = 30.5e^-8E-04x^	y = 24.9e^-0,002x^	y = 9.7e^-1E-03x^
Ab*	y = 30.4e^-9E-04x^	y = 22.7e^-0,002x^	y = 9.8e^-8E-04x^
B	y = 30.3e^-0,002x^	y = 24.1e^-0,002x^	y = 9.5e^-7E-04x^
C	y = 30.6e^-0,004x^	y = 23.9e^-0,003x^	y = 9.6e^-8E-04x^
D	y = 29.8e^-0,005x^	y = 23.6e^-0,004x^	y = 9.3e^-1E-03x^
E	y = 31.1e^-0,023x^	y = 24.3e^-0,02x^	y = 8.7e^-0,003x^

* Semen doses cooled with the Curves A, Aa and Ab had holding times of 6, 4 and 2 h at 22°C. All other samples (B, C, D and E) were cooled from 30°C to 5°C directly after filling, with no holding time.

### Experimental designs

#### Experiment 1

Semen doses prepared from one ejaculate of each boar (n = 8) were cooled to 5°C using five (A, B, C, D and E) different cooling rates and stored for 144 h (Fig 1). Samples cooled by Curve A, B, C, D and E had an initial cooling rate of 0.01, 0.06, 0.09, 0.14 and 0.65°C min^–1^, respectively, until reaching 25°C. Then, samples were cooled to 10°C at the rates of 0.03, 0.03, 0.05, 0.06 and 0.31°C min^–1^, respectively. Finally, samples were cooled to 5°C at a rate of 0.01°C min^–1^ (Curves A, B, C and D) and at 0.02°C min^–1^ (Curve E). Sperm analyses were performed after 24, 72 and 144 h of storage at 5°C. Bacterial species and bacterial counts (colony forming units (CFU) mL^–1^) were determined in the raw and extended semen of four randomly selected boars after 0, 24, 72 and 144 h storage and aerobic culture on blood agar for 48 h at 37°C.

#### Experiment 2

In this experiment using one ejaculate of each boar (n = 8), two intermediate cooling rates, named Aa and Ab (Fig 1), were added between Curves A and B (see Experiment 1) to test the effect of initial slow-to-moderate cooling between 30°C and 10°C. Samples cooled with Curves Aa and Ab had an initial cooling rate of 0.02°C min^–1^, during 2 and 4 h, respectively. After the holding times the samples reached temperatures of 25°C and 28°C, respectively. Samples were then cooled to 10°C at a rate of 0.04°C min^–1^. The final cooling from 10°C to 5°C was performed with cooling rates of 0.01°C min^–1^, for Curves Aa and Ab, respectively. Samples were cooled using Curves A and B, as previously described. Sperm analyses were performed after 24, 72 and 144 h storage at 5°C.

#### Experiment 3

Two aliquots of antibiotic-free AndroStar^®^ Premium extender were inoculated with *Escherichia coli* (*E*. *coli*; isolated from boar semen) at a concentration of 0.5 × 10^3^ CFU mL^–1^ in a final volume of 30 mL in a sterile Falcon tube. The tubes were placed in a cardboard box and stored in a temperature-controlled refrigerator at 5 ± 1°C. Samples were split into two groups with different holding times at 22°C, for 1 h and 6 h, respectively, and then cooled to 5°C. At 0, 3, 6, 24, 48, 72 and 144 h after bacterial inoculation, aliquots were plated on Luria Broth (LB) agar plates and incubated for 24 h at 37°C under aerobic conditions for enumeration of bacterial cells. The CFU mL^–1^ count was determined from two dilutions with two replicates per sample.

### Evaluation of sperm motility

To assess sperm motility, 2.0 mL aliquots were incubated at 38°C in a water bath under air contact. After 30 min of incubation, total sperm motility was determined by the computer‐assisted semen analysis (CASA) system, AndroVision^^®^^, version 1.1 (Minitüb, Germany), using four‐chamber slides (Leja, Nieuw Vennep, The Netherlands) with a depth of 20 μm. For each sample, six consecutive fields in the central axis of the chamber were recorded at a rate of 60 frames s^–1^ for each field using 100× magnification. At least 500 spermatozoa per sample were analyzed in all samples. Spermatozoa were evaluated as “motile” when their amplitude of lateral head displacement was greater than 1 μm and their curve line velocity was greater than 24.0 μm s^–1^.

### Flow cytometric assessment

Analyses were performed using a flow cytometer (CytoFLEX, Beckman Coulter, Krefeld, Germany) equipped with 405 nm and 488 nm solid-state lasers. Fluorescence signals of fluorescein isothiocyanate-conjugated peanut agglutinin (FITC-PNA), Yo-Pro1 and Fluo3-AM were gathered via a 525/40 nm band-pass filter. Hoechst 33342 signals were gathered via a Pb 450/45 nm band-pass filter, propidium iodide (PI) signals via a PC 5.5 690/50 nm band-pass filter, and Merocyanin 540 and JC-1 signals via a PE 585/42 nm long-pass filter. Non-DNA (Hoechst negative (neg)) particles were excluded and the sperm population was gated by referring to the expected forward- and side-scatter signals.

#### Evaluation of sperm membrane integrity

Integrity of sperm plasma membranes and acrosomes was analyzed at 24, 72 and 144 h using PI and FITC-PNA [14]. An aliquot of 50 μL of each diluted semen sample was transferred to 950 μL HEPES-buffered saline medium (HBS: 137 mM NaCl, 20 mM HEPES; 10 mM glucose; 2.5 mM KOH) + Bovine Serum Albumin (BSA: 1 mg mL^–1^) with adjusted pH (7.4 at 20°C) and osmolarity (300 ± 5 mOsmol kg^–1^) containing 5 μL Hoechst 33342 (final concentration 0.45 μg mL^–1^), 5 μL PI (final concentration 1 μg mL^–1^) and 5 μL FITC-PNA (final concentration 0.6 μg mL^–1^). After 5 min of incubation at 38°C in the dark, 10,000 events were analyzed. Spermatozoa were categorized as: (1) intact plasma membrane and acrosome (PI-neg/FITC-PNA-neg); (2) intact plasma membrane and damaged acrosome (PI-neg/FITC-PNA-positive (pos)); (3) damaged plasma membrane and intact acrosome (PI-pos/FITC-PNA-neg); (4) damaged plasma membrane and acrosome (PI-pos/FITC-PNA-pos). Sperm populations that exhibited intact plasma and acrosomal membranes were reported.

#### Evaluation of sperm membrane fluidity

The fluidity of the plasma membrane was assessed after staining the sperm samples with Merocyanine 540 and Yo-Pro-1 iodide [10]. An aliquot of 485 μL of each semen dose was incubated for 15 min at 38°C. After incubation, 5 μL Hoechst 33342 (final concentration 0.45 μg mL^–1^), 5 μL Yo-Pro-1 (final concentration 0.01 nM) and 5 μL Merocyanine 540 (final concentration 0.27 nM) were added to the semen samples and incubated for 15 min at 38°C. Thereafter, 50 μL of the sample was added to 950 μL of HBS solution and analyzed immediately. The spermatozoa were categorized as: (1) viable sperm with low plasma membrane fluidity (stable plasma membrane; Yo-Pro-1-neg/M-540-neg); (2) viable sperm with high plasma membrane fluidity (unstable plasma membrane; Yo-Pro-1 neg/M-540-pos); (3) dead sperm (Yo-Pro-1-pos). Viable spermatozoa with low plasma membrane fluidity were reported.

#### Evaluation of sperm mitochondrial membrane potential

The percentage of cells with a mitochondrial inner transmembrane potential (DCm) above a value of 80–100 mV was estimated after labeling samples with JC-1 and PI [15]. An aliquot of 1 mL of each semen dose was supplemented with 6 μL Hoechst 33342 (final concentration 0.9 μg mL^–1^), 12 μL PI (final concentration 12 μg mL^–1^) and 1 μL JC-1 (final concentration 1.53 nmol mL^–1^) and incubated for 15 min at 38°C. After incubation, 200 μL of the sample were added to 800 μL of HBS solution and immediately analyzed. Viable spermatozoa with a high mitochondrial membrane potential (hMMP; PI-neg and JC-1 pos) were reported.

#### Evaluation of sperm calcium influx

The response to capacitating conditions was evaluated by measurement of intracellular calcium [6, 16] in semen samples stored for 72 h. Spermatozoa were exposed to capacitating or non-capacitating conditions using two types of a Tyrode medium [17]. Tyrode capacitating medium (Tyrode A) consisted of 96 mM NaCl, 3.1 mM KCl, 0.4 mM MgSO_4_, 5 mM glucose, 15 mM NaHCO_3_, 2 mM CaCl_2_, 0.3 mM KH_2_PO_4_, 20 mM Hepes, 100 μg mL^–1^ gentamicin sulfate, 21.7 mM sodium lactate, 1 mM sodium pyruvate, 3 mg BSA mL^–1^, 100 μg gentamicin mL^–1^ and 20 μg mL^–1^ phenol red. In non-capacitating Tyrode control medium (Tyrode C) NaHCO_3_ was omitted and the NaCl content was increased to 111 mM. Both media were adjusted to a pH of 7.6 at 20°C and a final osmolarity of 300 ± 5 mOsmol kg^–1^. Prior to analysis, 2 μL of PI (final concentration 2 μg mL^–1^) and 2 μL of Hoechst (final concentration 0.6 μg mL^–1^) were added to 946 μL of both Tyrode media and incubated at 38°C under CO_2_ (Tyrode A) or under air contact (Tyrode C), in the dark. In parallel, aliquots of 2 mL of extended semen were supplemented with 2.5 μL Hoechst 33342 (final concentration 0.75 μg mL^–1^) and 2 μL Fluo-3-AM (final concentration 1 μM) and incubated for 30 min at room temperature in the dark. After incubation, 50 μL of each sample was added to 950 μL of Tyrode A and Tyrode C. Samples were assessed after 3 and 60 min of incubation at 38°C under CO_2_ (Tyrode A) or under air contact (control).

The analysis focused on the evaluation of the specific response to bicarbonate of viable spermatozoa [10, 16]. Therefore, the percentage of viable sperm with low intracellular calcium content (PI-neg/Fluo-3-neg) and viable sperm with high calcium content (PI-neg/Fluo-3-pos), as well as percentages of PI-pos sperm, were determined. The response to Tyrode A was calculated as the difference between the percentage of spermatozoa at 60 min and at the onset of incubation (Tyrode A Δ60–3) in the sperm populations. The same procedure was applied to the sperm populations in the control medium (control Δ60–3). The specific response to the capacitating stimulus (bicarbonate) was then calculated as the difference between the responses in the low intracellular calcium content in viable sperm in capacitating Tyrode and in control medium.

#### Statistical analyses

Data analysis was performed using Statistical Analysis System software (SAS Enterprise Guide, version 7.1; SAS Inst. Inc., Cary, NC). Experimental data were checked for normal distribution followed by pairwise comparisons using Student’s t-test and two-factorial analysis of variance (ANOVA) with repeated measurements. Boars were considered as a random effect in the models used. When the ANOVA revealed a significant difference among groups, comparisons using post hoc Tukey–Kramer test was performed. Differences among means were considered significant at P < 0.05. Data are presented as mean ± standard deviation (SD).

## Results

### Experiment 1

**Fig 2:**
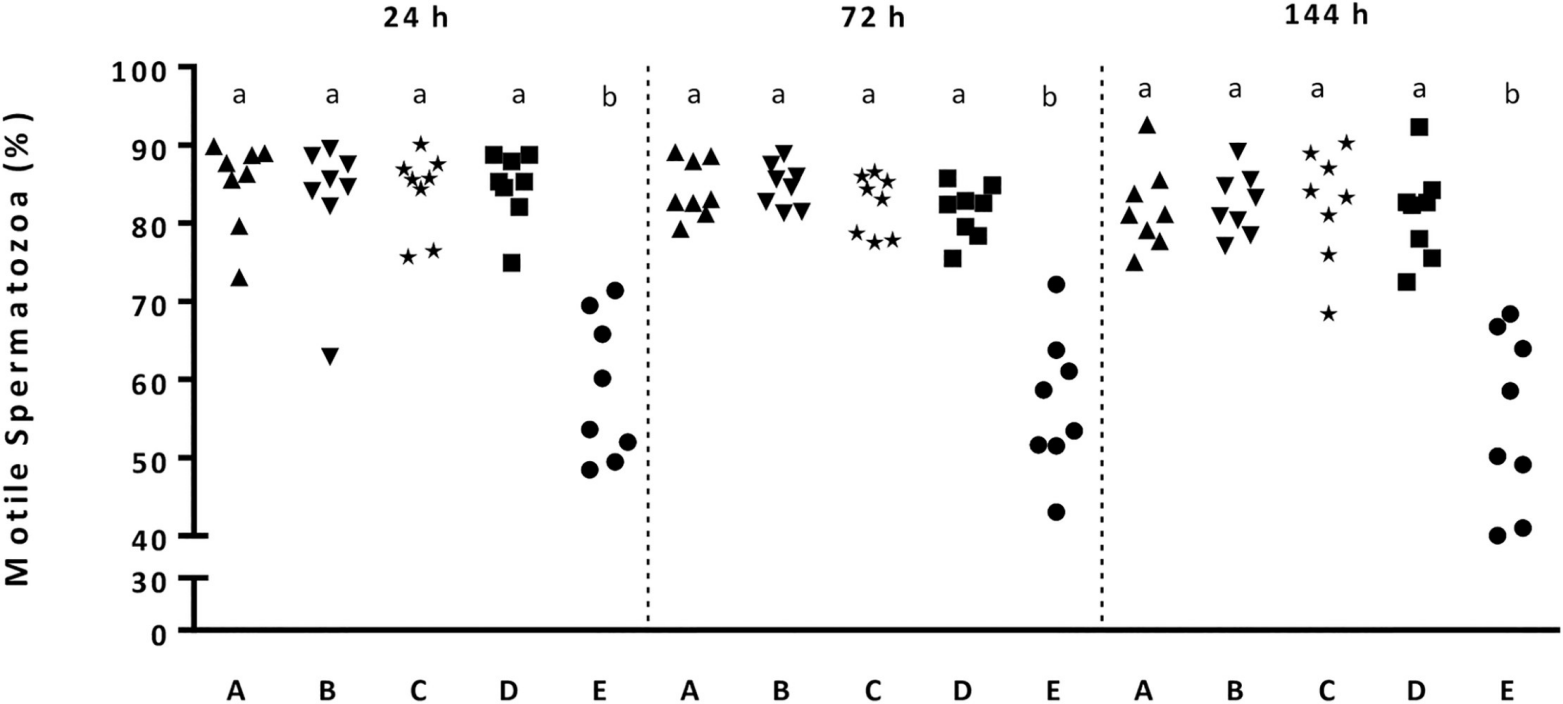
Scatterplots showing sperm motility in semen samples (n = 8) cooled at different cooling rates (A, B, C, D and E–Experiment 1) at 24, 72 and 144 h storage at 5°C. a,b: values differ significantly (P < 0.01).

**Fig 3:**
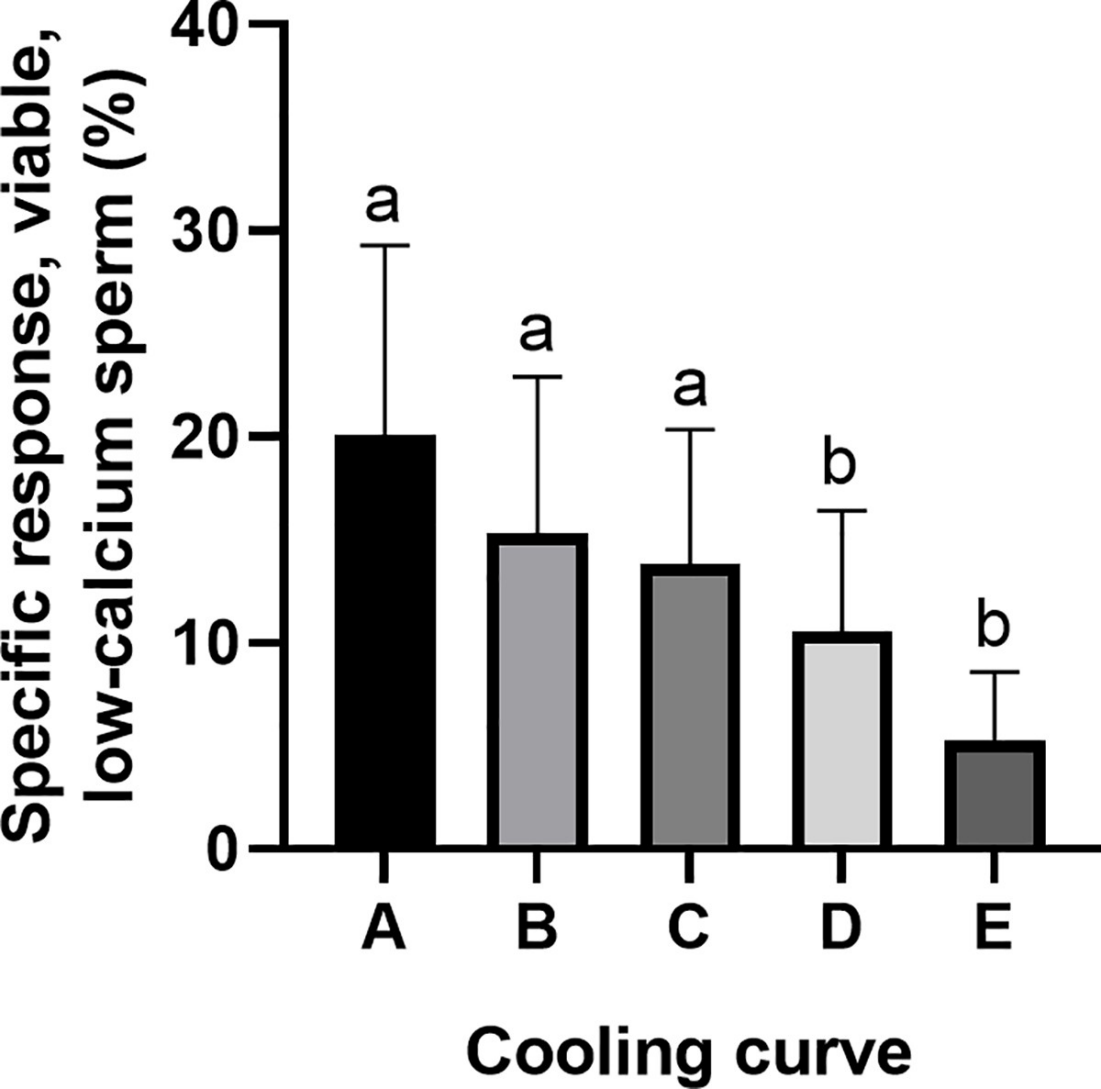
Specific response to capacitating conditions calculated as the difference in the responsiveness to incubation conditions in Tyrode (Tyrode A Δ60 – 3) and control (control Δ60 – 3) medium for the population of viable sperm with low calcium content (PI-neg./Fluo-3-neg.) in semen samples cooled with different cooling rates (A, B, C, D and E–Experiment 1) to 5°C and stored for 72 h. Data are shown as mean ± SD (n = 8 boars). a,b: values differ significantly (P < 0.05).

The cooling rate influenced (P < 0.01) sperm motility, membrane integrity, membrane fluidity and mitochondria membrane potential, whereas storage time did not show an influence. Sperm quality traits were at a high level at 144 h storage time in samples subjected to cooling rates A–D. Mean values of total motility at 144 h for cooling rates A–D were 82.0 ± 5.1%, 82.5 ± 3.8%, 82.4 ± 6.8% and 81.3 ± 7.7%, respectively (P > 0.05). The means of membrane intact spermatozoa, spermatozoa with low membrane fluidity and hMMP are shown in Table 2. In samples submitted to cooling rate E, the percentages of motile spermatozoa (Fig 2) and of membrane intact (PI-neg/FITC-PNA neg) spermatozoa were lower (P < 0.01) compared to samples cooled at the other cooling rates (Table 2) after 24, 72 and 144 h storage. Accordingly, the percentage of viable spermatozoa with low membrane fluidity was lower (P < 0.01) at all time points in doses cooled with Curve E compared to all others. At 72 h of storage, the doses cooled with Curve D had lower (P < 0.01) percentages of viable spermatozoa with low membrane fluidity (73.6 ± 7.7%) compared to B (79.4 ± 4.4%—Table 2). The percentage of spermatozoa with hMMP at 24 h was lower (P < 0.01) when doses were cooled with Curve E compared to those cooled with Curves A, B, C and D. The specific response to the capacitating stimulus bicarbonate was lower (P < 0.01) for the doses cooled with cooling rates D and E compared to cooling rates A, B and C (Fig 3).

**Table 2:** Spermatozoa with intact plasma membrane (viable) and acrosome (PI-neg and FITC-PNA-neg), viable spermatozoa with low plasma membrane fluidity (Yo-Pro1-neg and M-540-neg) and viable spermatozoa with high mitochondrial membrane potential in semen samples extended in antibiotic-free AndroStar^®^ Premium and submitted to cooling rates A, B, C, D and E (Experiment 1; c.f. Table 1) before storage at 5°C. Data are presented as mean ± SD (n = 8 boars).

		Storage time		
Parameter	Cooling curve	24 h	72 h	144 h
Viable spermatozoa (%) with intact acrosomes (PI-neg and FITC-neg)	A	81.9 ± 5.6^a^	82.8 ± 5.3^a^	83.8 ± 3.3^a^
	B	82.7 ± 4.6^a^	84.7 ± 3.0^a^	85.1 ± 2.9^a^
	C	81.8 ± 5.2^a^	82.7 ± 3.5^a^	83.0 ± 3.2^a^
	D	79.0 ± 4.7^a^	80.7 ± 5.0^a^	80.8 ± 3.8^a^
	E	58.8 ± 9.6^b^	59.0 ± 10.1^b^	55.5 ± 9.4^b^
Viable spermatozoa with high mitochondrial membrane potential (PI-neg and JC-1-pos) (%)	A	94.2 ± 2.0^a^	95.2 ± 2.0^a^	94.2 ± 0.8^a^
	B	94.1 ± 1.8^a^^b^	94.3 ± 1.4^a^	94.0 ± 1.5^a^
	C	94.0 ± 2.5^a^^b^	94.3 ± 2.2^a^	93.8 ± 1.4^a^
	D	93.7 ± 1.5^a^^b^	93.1 ± 1.4^a^	92.8 ± 1.6^a^
	E	92.0 ± .2.6^b^	90.0 ± 2.5^b^	88.1 ± 4.2^b^
Viable spermatozoa (%) with low membrane fluidity (Yo-Pro-1-neg and M-540-neg)	A	75.4 ± 6.2^a^	76.9 ± 5.8^a^^b^	76.3 ± 6.6^a^
	B	76.2 ± 5.2^a^	79.4 ± 4.4^a^	79.3 ± 6.5^a^
	C	76.1 ± 4.6^a^	76.9 ± 5.9^a^^b^	77.1 ± 5.1^a^
	D	71.2 ± 5.0^a^	73.6 ± 7.7^b^	75.9 ± 6.1^a^
	E	55.8 ± 12.0^b^	49.3 ± 11.7^c^	46.4 ± 10.4^b^

^a–c^ indicates significant differences within a column (P < 0.05). FITC-PNA: fluorescein isothiocyanate-conjugated peanut agglutinin; PI: propidium iodide; M-540: Merocyanine 540.

**Fig 4:**
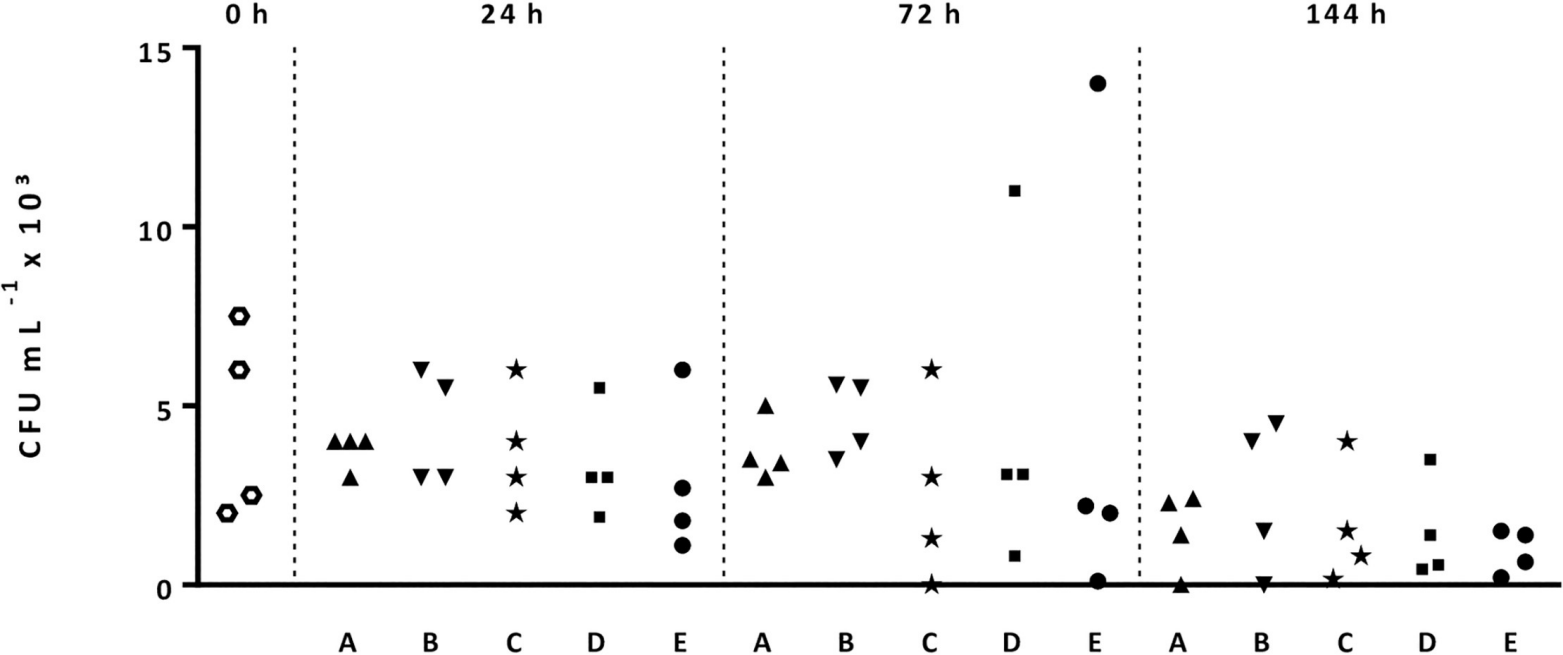
Scatterplots (n = 4 boars) showing bacteria counts (CFU mL^–1^) in semen extended in antibiotic-free AndroStar^®^ Premium and cooled with different cooling rates (A, B, C, D and E–Experiment 1) before storage at 5°C.

Bacterial counts in raw semen ranged from 1.7 × 10^4^ to 8.5 × 10^4^ CFU mL^–1^ (4.1 × 10^4^ ± 2.7 × 10^4^ CFU mL^–1^), being identified Gram-negative (*E*. *coli*, *Pseudomonas aeruginosa*) and Gram-positive (*Staphylococcus* spp., *Streptococcus* spp., *Bacillus* spp.) bacteria. Microbiology results in extended semen doses immediately after dilution (0 h), at 24, 72 and 144 h storage are presented in Fig 4. The bacterial load in diluted sperm at 0 h storage ranged from 1.0 × 10^1^ to 7.5 × 10^3^ CFU mL^–1^. At 72 h, bacterial load in two samples, one cooled with Curve A and one cooled with Curve B, increased to 1.1 × 10^4^ and 1.4 × 10^4^ CFU mL^–1^, respectively (Fig 3). At 144 h, all samples revealed CFU < 6.0 × 10^3^ mL^–1^.

### Experiment 2

Sperm motility (range between cooling rates and storage time, respectively: 86.1 ± 1.6 to 91.3 ± 3.0) remained at a high level throughout storage and was not influenced by the cooling rate. Likewise, the cooling rate did not influence membrane integrity and mitochondria membrane potential (Table 3). A significant effect of cooling rate and time of storage was observed for the percentage of sperm with low membrane fluidity (P = 0.02; P = 0.01, respectively). The percentage of viable spermatozoa with low membrane fluidity was lower (P < 0.01) after 24 h storage of spermatozoa cooled by A (75.9 ± 4.2) compared to spermatozoa cooled by B (79.4 ± 3.9%). However, sperm quality traits were at a high level after 144 h storage in samples subjected to cooling rates A, Aa, Ab and B (Table 3). The specific response to the capacitating stimulus bicarbonate did not differ for the cooling rates A, Aa, Ab and B (range 12.8 ± 5.6 to 15.1 ± 4.9).

**Table 3:** Spermatozoa with intact plasma membrane (viable) and acrosome (PI-neg and FITC-PNA-neg), viable spermatozoa with low plasma membrane fluidity (Yo-Pro-1-neg and M-540-neg) and viable spermatozoa with high mitochondrial membrane potential in semen samples extended in antibiotic-free AndroStar^®^ Premium and submitted to cooling rates A, Aa, Ab and B (Experiment 2) before storage at 5°C. Data are presented as mean ± SD (n = 8 boars).

		Storage time		
Parameter	Cooling curve	24 h	72 h	144 h
Viable spermatozoa (%) with intact acrosomes (PI-neg and FITC-neg)	A	86.6 ± 0.7^a^	84.2 ± 2.6^a^	86.1 ± 2.8^a^
	Aa	87.1 ± 1.7^a^	85.4 ± 1.8^a^	85.8 ± 2.1^a^
	Ab	87.2 ± 2.0^a^	86.2 ± 1.6^a^	86.8 ± 2.8^a^
	B	87.0 ± 2.1^a^	85.2 ± 3.2^a^	86.5 ± 3.1^a^
Viable spermatozoa (%) with low membrane fluidity (Yo-Pro-1-neg and M-540-neg)	A	75.9 ± 4.2^a^	77.1 ± 5.8^a^	73.6 ± 11.2^a^
	Aa	76.6 ± 5.0^a^^b^	77.5 ± 6.2^a^	74.4 ± 10.8^a^
	Ab	78.0 ± 3.9^a^^b^	79.4 ± 6.0^a^	76.6 ± 9.2^a^
	B	79.4 ± 3.9^b^	79.5 ± 5.8^a^	77.0 ± 7.7^a^
Viable spermatozoa (%) with high mitochondrial membrane potential (PI-neg, JC-1-pos)	A	95.0 ± 1.2^a^	94.5 ± 0.8^a^	94.5 ± 1.5^a^
	Aa	95.6 ± 1.5^a^	94.5 ± 0.9^a^	94.7 ± 1.3^a^
	Ab	95.1 ± 1.0^a^	94.4 ± 1.1^a^	94.8 ± 1.0^a^
	B	94.8 ± 1.1^a^	94.2 ± 0.7^a^	94.9 ± 1.0^a^

^a,b^ indicates significant differences within a column (P < 0.05). FITC-PNA: fluorescein isothiocyanate-conjugated peanut agglutinin; PI: propidium iodide; M-540: Merocyanine 540.

### Experiment 3

Following inoculation of semen-free AndroStar^®^ Premium extender with 0.5 × 10^3^ CFU mL^–1^ (*E*. *coli*), samples exposed to a short holding time of 1 h at RT (22°C) prior to cooling contained 1.0 × 10^3^ CFU mL^–1^, while samples held for 6 h at RT contained 2.4 × 10^3^ CFU mL^–1^ (Table 4). Bacterial counts were reduced in both samples after cooling, with 0.7 × 10^3^ CFU mL^–1^ (1 h holding time) and 2.2 x10^3^ CFU mL^–1^ (6 h holding time) respectively, after 24 h. Continuous storage at 5°C for 144 h resulted in an overall decrease of the bacterial load to <1 × 10^3^ CFU mL^–1^. In samples exposed to a long holding time of 6 h before cooling, bacterial counts did not fall below the initial inoculation number.

**Table 4:** Bacterial counts (CFU mL^–1^) in antibiotic-free AndroStar^®^ Premium extender inoculated with 0.5 × 10^3^ *E*. *coli* and held either for 1 h or 6 h at room temperature (RT, 22°C) before storage at 5°C. Data are single values and means of two replicates (n = 2, Experiment 3).

	Time of storage (h) at 5°C									
	0 h		6 h		24 h		72 h		144 h	
Time at RT	RT1	RT6	RT1	RT6	RT1	RT6	RT1	RT6	RT1	RT6
Sample 1	455	773	1045	2682	727	2591	591	1818	409	955
Sample 2	636	364	1000	2045	682	1773	136	2227	182	773
Mean	545	568	1023	2364	705	2182	364	2023	295	864

RT1: Samples were held for 1 hour at RT (22°C) before storage at 5°C.

RT6: Samples were held for 6 hours at RT (22°C) before storage at 5°C.

## Discussion

In the present study, an appropriate cooling-rate frame for extended boar semen designed for antibiotic-free storage was established, from 0.01 to 0.09°C min^‑1^ in the temperature zone from 30 to 25°C, followed by 0.02 to 0.06°C min^–1^ to 10°C and 0.01 to 0.02°C min^‑1^ to the final storage temperature. The respective temperature graphs are presented by the cooling rates Aa, Ab, B and C in this article. In cryopreservation protocols, cooling rates ranging from 0.08 to 1.5°C min –1 within a temperature zone of 15°C to 5°C are considered effective for chilling boar spermatozoa prior to freezing [18, 19]. Recommendations of cooling rates between 30°C and 5°C and subsequent liquid storage at this temperature have been lacking until now. Freshly ejaculated sperm react sensitively to fast temperature decrease, as demonstrated by the lipid phase transition and fluidity decrease of plasma head membranes occurring between 30°C and 10°C in boar spermatozoa [10, 20]. Loss of motility and membrane integrity caused by rapid initial cooling becomes increasingly apparent during conventional semen storage at 17°C [21] and might be even more pronounced at 5°C. Consequently, slow cooling with holding times at RT were recommended before liquid storage at 17°C [21]. Here we provide the first evidence for an optimal cooling rate from 30°C to 5°C for boar spermatozoa, considering both sperm quality traits and bacterial growth. The moderate and slow cooling rates between Curves A and C yielded consistently high sperm quality throughout long-term semen storage. Notably, a protecting extender was used, which had previously proved to preserve sperm characteristics and fertility during storage at 5°C [6]. Fast cooling by Curve E yielded highly variable motility values already by 24 h of storage indicating that boars might respond differently to chilling stress, similarly as described for the inter-boar variability in the response to sperm cryopreservation [22].

Loss of sperm motility indicates chilling and storage damage in spermatozoa, and thus motility is the most important indicator used in boar AI centers, with a minimum threshold of 65% for usable semen [1]. In our study, only those semen doses cooled rapidly (cooling Curve E) did not reach threshold values of sperm motility and had already displayed a significant loss of membrane integrity after 24 h storage. Chilling injury at a sublethal level was evaluated in plasma membrane intact (“viable”) spermatozoa by flow cytometric assessment of plasma membrane fluidity, MMP, and the response to the capacitation stimulant bicarbonate. Confirming earlier studies that reported an influence of hypothermic liquid storage on membrane fluidity [10, 12], we observed a clear decrease in viable sperm with low membrane fluidity in semen samples with the fastest cooling rate (Curve E) compared to samples cooled more slowly (A, B and C). The MMP was also affected by this cooling rate, thus confirming a previous study that reported a decrease in MMP and oxidoreductive capability of boar sperm stored at 5°C [23]. The chilling-induced loss of mitochondrial function may contribute to the lower ATP concentration reported in chilled boar spermatozoa [24], which could cause metabolic disruption; specifically, the decrease of motility [25] as observed here. Perturbance of mitochondrial function is probably connected to a loss of coordinated transfer of ions and other solutes through the plasma membrane. This may also explain a chilling-induced increase in cytosolic calcium ions leading to premature cell destabilization and a reduced specific response to the capacitation stimulus bicarbonate [26].

Assessment of calcium influx under capacitation conditions, particularly the response to bicarbonate, is an established measure for chilling injury in hypothermically stored boar semen [10, 26] and proved to be most sensitive in the present study for mirroring the effects of cooling velocity on sperm function. Rapid cooling, according to cooling Curves D and E, resulted in a lower response to bicarbonate, indicating that the loss of essential sperm function may occur before sperm motility or viability are affected. Moreover, it is important to consider that plasma membrane alterations could lead to a higher permeability to calcium due to the disruption of the ability to achieve normal capacitation [27]. The ability to respond to capacitation signals depends on complex signal transduction pathways [28], and a decreased *in vitro* responsiveness is associated with subfertility in normospermic boars [29].

As outlined above, from the perspective of sperm function, slow cooling seems to be advantageous; however, the risk of bacterial growth during delayed cooling needs to be considered. This general notion is supported by the results of the present study, which showed higher bacterial counts of *E*. *coli* in the inoculated sperm-free semen extender held for 6 h at 22°C compared to samples held for only 1 h (Experiment 3). The presence of microbial contaminants, mostly identified as Gram-negative bacteria belonging to the *Enterobacteriaceae* family [4], may impair sperm viability and fertility in a dose-dependent manner. However, the experimental inoculation of semen with 10^4^ CFU mL^–1^ of *E*. *coli* has been found not to influence the quality of human spermatozoa [30], and adverse effects on sperm traits were reported for boar semen starting with minimum values of 10^7^ or 10^8^ CFU mL^–1^ of *E*. *coli* [31, 32]. In previously studies [33], it was reported that *E*. *coli* counts of 3.5 × 10^3^ CFU mL^‑1^ affected litter size, several dose-response studies showed that the detrimental effects of bacteria (*E*. *coli*, *Cl*. *perfringens*, *Ps*. *aeruginosa*) were only apparent at the highest concentrations, ranging between 10^6^ and 10^8^ CFU mL^–1^ [31, 34–36]. Some authors propose that the sperm/bacteria ratio of 1:1 presents the tolerance limit for sperm injury [30, 37, 38], which would translate into an upper limit of 2–3 × 10^7^ CFU mL^–1^ in extended boar semen. In our previous study [6], in which slow cooling according to Curve A was applied to antibiotic-free semen doses, fertility was not affected in the presence of up to 7.6 × 10^3^ CFU mL^–1^. In agreement with previous reports [37, 39], all ejaculates included in the present study were positive for the presence of bacteria, confirming that the collection of sterile semen is virtually impossible. More bacteria were found in semen samples with slower cooling rates (Curves A, B), but bacterial counts (up to 10^4^ CFU mL^–1^) did not exceed the proposed limits of sperm/bacteria ratios as described above. Due to the mesophilic character of the typical semen contaminants, the bacteriostatic effect of hypothermic storage increased with ongoing storage time, thus favoring the use of stored semen rather than freshly diluted ejaculates.

It is important to consider that semen doses prepared for transport to the research laboratory or to the farm might differ in their cooling velocity depending on whether they were placed in the center or at the margin of tube bundles. The volumes of semen doses (commonly in tubes or bags with 30–100 mL of extended semen), packing units and types of packaging may vary, thus pointing to the necessity to control cooling rates at representative positions of a given semen package. For transfer of the current findings into practice, it must be ensured that all semen doses in a package reach a cooling velocity that lies within the recommended frame reported in this study.

## Conclusions

In conclusion, moderate cooling rates within the frame presented by cooling-rate Curves Aa and C are recommended for antibiotic-free storage of boar semen at 5°C. With the cooling protocols described here, an appropriate cooling regime can easily be implemented into research laboratories or AI centers, thus allowing instant use of the hypothermic storage concept. Further studies are encouraged to assess the potential of hypothermic semen preservation with the aim of reducing the use of antibiotics in artificial insemination of pigs.

## Supporting information

S1 File (XLSX)
